# Phenyl quinoxalin-2-yl ether

**DOI:** 10.1107/S1600536808026809

**Published:** 2008-08-23

**Authors:** Nor Duha Hassan, Hairul Anuar Tajuddin, Zanariah Abdullah, Seik Weng Ng

**Affiliations:** a Department of Chemistry, University of Malaya, 50603 Kuala Lumpur, Malaysia

## Abstract

The aromatic ring systems in the title compound, C_14_H_10_N_2_O, form a dihedral angle of 63.8 (1)°, resulting in an opening up of the ether-O atom angle to 118.2 (1)°.

## Related literature

The title compound exhibits fluorescence; see: Abdullah (2005 ▶); Kawai *et al.* (2001 ▶); Mohd Salleh *et al.* (2007 ▶). For the only previously reported structural example of a quinoxalin­oxy compound, see: Csikós *et al.* (1999 ▶).
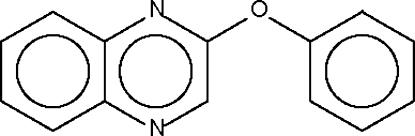


## Experimental

### 

#### Crystal data

C_14_H_10_N_2_O
*M* *_r_* = 222.24
Monoclinic, 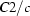

*a* = 18.175 (2) Å
*b* = 6.6589 (8) Å
*c* = 19.488 (2) Å
β = 112.937 (2)°
*V* = 2172.1 (5) Å^3^
*Z* = 8
Mo *K*α radiation
μ = 0.09 mm^−1^
*T* = 100 (2) K
0.30 × 0.20 × 0.05 mm

#### Data collection

Bruker SMART APEX diffractometer
Absorption correction: none
6018 measured reflections
2478 independent reflections
1909 reflections with *I* > 2σ(*I*)
*R*_int_ = 0.026

#### Refinement

*R*[*F* ^2^ > 2σ(*F* ^2^)] = 0.039
*wR*(*F* ^2^) = 0.104
*S* = 1.07
2478 reflections
154 parameters
H-atom parameters constrained
Δρ_max_ = 0.27 e Å^−3^
Δρ_min_ = −0.27 e Å^−3^

### 

Data collection: *APEX2* (Bruker, 2007 ▶); cell refinement: *SAINT* (Bruker, 2007 ▶); data reduction: *SAINT*; program(s) used to solve structure: *SHELXS97* (Sheldrick, 2008 ▶); program(s) used to refine structure: *SHELXL97* (Sheldrick, 2008 ▶); molecular graphics: *X-SEED* (Barbour, 2001 ▶); software used to prepare material for publication: *publCIF* (Westrip, 2008 ▶).

## Supplementary Material

Crystal structure: contains datablocks global, I. DOI: 10.1107/S1600536808026809/tk2297sup1.cif

Structure factors: contains datablocks I. DOI: 10.1107/S1600536808026809/tk2297Isup2.hkl

Additional supplementary materials:  crystallographic information; 3D view; checkCIF report

## Figures and Tables

**Fig. 1.**
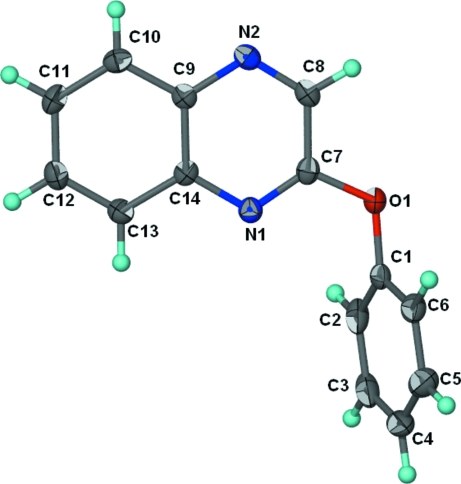
Thermal ellipsoid plot (Barbour, 2001) of (I) drawn at the 70% probability level; hydrogen atoms are drawn as spheres of arbitrary radius.

## supplementary crystallographic information

### Comment

The title compound (I) belongs to a class of compounds that exhibits fluorescence (Abdullah, 2005; Kawai *et al.*, 2001; Mohd Salleh *et al.*, 2007). In the crystal structure, the two aromatic systems aligned at 63.8 (1) °; these open up the angle at the oxygen atom to 118.2 (1) ° (Fig. 1).

In the structural literature, there is only one example of a quinoxalinoxy compound, 2(1*H*-quinoxalone *O*-(2'-quinoxalinyl)hydroxylamine, which exists in two colored forms (Csikós *et al.*, 1999).

### Experimental

Phenol (0.47 g, 5 mmol) was dissolved in a small volume of water containing potassium hydroxide (0.20 g, 5 mmol). The mixture was heated to remove the water to give a brown compound. The compound and 2-chloroquinoxaline (0.82 g, 5 mmol) were heated in THF (15 ml) for 8 h. The mixture was in 1 N sodium hydroxide; the aqueous solution was extracted with dichloromethane. The organic phase was dried over sodium sulfate. Evaporation of the solvent gave a yellow product, which was was washed with chloroform to remove impurities. Crystals were obtained upon recrystallization from an ethyl acetate/hexane mixture of (I).

### Refinement

Carbon-bound H-atoms were placed in calculated positions (C—H 0.95 Å) and were included in the refinement in the riding model approximation, with *U*(H) fixed at 1.2*U*(C).

### Crystal data

**Table:**

C_14_H_10_N_2_O	*F*_000_ = 928
*M**_r_* = 222.24	*D*_x_ = 1.359 Mg m^−^^3^
Monoclinic, *C*2/*c*	Mo *K*α radiation λ = 0.71073 Å
Hall symbol: -C 2yc	Cell parameters from 1614 reflections
*a* = 18.175 (2) Å	θ = 2.4–28.2º
*b* = 6.6589 (8) Å	µ = 0.09 mm^−^^1^
*c* = 19.488 (2) Å	*T* = 100 (2) K
β = 112.937 (2)º	Prism, colorless
*V* = 2172.1 (5) Å^3^	0.30 × 0.20 × 0.05 mm
*Z* = 8	

### Data collection

**Table:**

Bruker SMART APEX diffractometer	1909 reflections with *I* > 2σ(*I*)
Radiation source: fine-focus sealed tube	*R*_int_ = 0.026
Monochromator: graphite	θ_max_ = 27.5º
*T* = 100(2) K	θ_min_ = 2.3º
ω scans	*h* = −23→16
Absorption correction: None	*k* = −8→8
6018 measured reflections	*l* = −23→25
2478 independent reflections	

### Refinement

**Table:**

Refinement on *F*^2^	Secondary atom site location: difference Fourier map
Least-squares matrix: full	Hydrogen site location: inferred from neighbouring sites
*R*[*F*^2^ > 2σ(*F*^2^)] = 0.039	H-atom parameters constrained
*wR*(*F*^2^) = 0.104	*w* = 1/[σ^2^(*F*_o_^2^) + (0.0479*P*)^2^ + 0.7132*P*] where *P* = (*F*_o_^2^ + 2*F*_c_^2^)/3
*S* = 1.07	(Δ/σ)_max_ = 0.001
2478 reflections	Δρ_max_ = 0.27 e Å^−^^3^
154 parameters	Δρ_min_ = −0.27 e Å^−^^3^
Primary atom site location: structure-invariant direct methods	Extinction correction: none

### Fractional atomic coordinates and isotropic or equivalent isotropic displacement parameters (Å^2^)

**Table:**

	*x*	*y*	*z*	*U*_iso_*/*U*_eq_	
O1	0.68658 (5)	0.68613 (14)	0.55215 (5)	0.0196 (2)	
N1	0.59181 (6)	0.76551 (15)	0.59977 (6)	0.0166 (2)	
N2	0.47638 (6)	0.72591 (16)	0.45219 (6)	0.0176 (2)	
C1	0.74734 (7)	0.7128 (2)	0.62327 (7)	0.0178 (3)	
C2	0.75301 (8)	0.5866 (2)	0.68141 (8)	0.0242 (3)	
H2	0.7143	0.4846	0.6749	0.029*	
C3	0.81628 (9)	0.6118 (2)	0.74933 (8)	0.0291 (4)	
H3	0.8209	0.5269	0.7900	0.035*	
C4	0.87301 (9)	0.7599 (2)	0.75850 (8)	0.0293 (4)	
H4	0.9163	0.7763	0.8053	0.035*	
C5	0.86637 (8)	0.8838 (2)	0.69930 (8)	0.0267 (3)	
H5	0.9053	0.9849	0.7055	0.032*	
C6	0.80290 (8)	0.8608 (2)	0.63088 (7)	0.0208 (3)	
H6	0.7980	0.9456	0.5901	0.025*	
C7	0.60973 (7)	0.71759 (18)	0.54400 (7)	0.0167 (3)	
C8	0.55218 (8)	0.69640 (19)	0.46909 (7)	0.0180 (3)	
H8	0.5698	0.6599	0.4309	0.022*	
C9	0.45400 (7)	0.77709 (18)	0.50983 (7)	0.0156 (3)	
C10	0.37309 (8)	0.81243 (19)	0.49536 (7)	0.0184 (3)	
H10	0.3341	0.8006	0.4460	0.022*	
C11	0.35029 (8)	0.86377 (19)	0.55218 (7)	0.0202 (3)	
H11	0.2955	0.8882	0.5420	0.024*	
C12	0.40760 (8)	0.8806 (2)	0.62563 (7)	0.0204 (3)	
H12	0.3912	0.9162	0.6647	0.025*	
C13	0.48681 (8)	0.8460 (2)	0.64120 (7)	0.0189 (3)	
H13	0.5249	0.8564	0.6910	0.023*	
C14	0.51180 (7)	0.79510 (18)	0.58369 (7)	0.0157 (3)	

### Atomic displacement parameters (Å^2^)

**Table:**

	*U*^11^	*U*^22^	*U*^33^	*U*^12^	*U*^13^	*U*^23^
O1	0.0157 (5)	0.0261 (5)	0.0182 (5)	0.0021 (4)	0.0079 (4)	−0.0013 (4)
N1	0.0167 (6)	0.0160 (5)	0.0179 (5)	−0.0007 (4)	0.0077 (4)	−0.0009 (4)
N2	0.0214 (6)	0.0154 (5)	0.0166 (5)	−0.0008 (4)	0.0079 (4)	−0.0009 (4)
C1	0.0158 (6)	0.0218 (6)	0.0172 (6)	0.0052 (5)	0.0079 (5)	−0.0004 (5)
C2	0.0238 (7)	0.0257 (7)	0.0295 (7)	0.0071 (6)	0.0174 (6)	0.0064 (6)
C3	0.0297 (8)	0.0404 (9)	0.0233 (7)	0.0181 (7)	0.0169 (6)	0.0118 (6)
C4	0.0222 (7)	0.0439 (9)	0.0184 (7)	0.0127 (7)	0.0045 (6)	−0.0019 (6)
C5	0.0208 (7)	0.0291 (7)	0.0277 (7)	0.0011 (6)	0.0067 (6)	−0.0045 (6)
C6	0.0196 (7)	0.0228 (7)	0.0215 (7)	0.0036 (5)	0.0097 (6)	0.0029 (5)
C7	0.0161 (6)	0.0148 (6)	0.0208 (6)	−0.0003 (5)	0.0090 (5)	0.0005 (5)
C8	0.0221 (7)	0.0164 (6)	0.0176 (6)	−0.0006 (5)	0.0102 (5)	−0.0007 (5)
C9	0.0182 (6)	0.0120 (6)	0.0170 (6)	−0.0008 (5)	0.0074 (5)	−0.0006 (5)
C10	0.0177 (7)	0.0159 (6)	0.0185 (6)	−0.0008 (5)	0.0037 (5)	0.0000 (5)
C11	0.0149 (6)	0.0189 (6)	0.0270 (7)	0.0001 (5)	0.0084 (5)	0.0006 (5)
C12	0.0220 (7)	0.0213 (6)	0.0215 (6)	−0.0001 (5)	0.0123 (6)	−0.0019 (5)
C13	0.0196 (7)	0.0205 (6)	0.0168 (6)	−0.0005 (5)	0.0072 (5)	−0.0009 (5)
C14	0.0152 (6)	0.0133 (6)	0.0184 (6)	−0.0006 (5)	0.0062 (5)	0.0003 (5)

### Geometric parameters (Å, °)

**Table:**

O1—C7	1.3591 (15)	C5—H5	0.9500
O1—C1	1.4068 (15)	C6—H6	0.9500
N1—C7	1.2904 (17)	C7—C8	1.4335 (17)
N1—C14	1.3771 (16)	C8—H8	0.9500
N2—C8	1.3006 (17)	C9—C10	1.4051 (18)
N2—C9	1.3782 (17)	C9—C14	1.4182 (17)
C1—C6	1.3769 (19)	C10—C11	1.3685 (19)
C1—C2	1.3821 (18)	C10—H10	0.9500
C2—C3	1.384 (2)	C11—C12	1.4086 (18)
C2—H2	0.9500	C11—H11	0.9500
C3—C4	1.387 (2)	C12—C13	1.3703 (18)
C3—H3	0.9500	C12—H12	0.9500
C4—C5	1.385 (2)	C13—C14	1.4048 (18)
C4—H4	0.9500	C13—H13	0.9500
C5—C6	1.3903 (18)		
			
C7—O1—C1	118.16 (10)	O1—C7—C8	114.37 (11)
C7—N1—C14	115.75 (11)	N2—C8—C7	121.57 (12)
C8—N2—C9	116.80 (11)	N2—C8—H8	119.2
C6—C1—C2	121.99 (12)	C7—C8—H8	119.2
C6—C1—O1	117.24 (11)	N2—C9—C10	119.73 (11)
C2—C1—O1	120.65 (12)	N2—C9—C14	120.73 (12)
C1—C2—C3	118.61 (14)	C10—C9—C14	119.54 (12)
C1—C2—H2	120.7	C11—C10—C9	120.18 (12)
C3—C2—H2	120.7	C11—C10—H10	119.9
C2—C3—C4	120.54 (13)	C9—C10—H10	119.9
C2—C3—H3	119.7	C10—C11—C12	120.33 (12)
C4—C3—H3	119.7	C10—C11—H11	119.8
C5—C4—C3	119.82 (13)	C12—C11—H11	119.8
C5—C4—H4	120.1	C13—C12—C11	120.57 (12)
C3—C4—H4	120.1	C13—C12—H12	119.7
C4—C5—C6	120.25 (14)	C11—C12—H12	119.7
C4—C5—H5	119.9	C12—C13—C14	120.16 (12)
C6—C5—H5	119.9	C12—C13—H13	119.9
C1—C6—C5	118.80 (13)	C14—C13—H13	119.9
C1—C6—H6	120.6	N1—C14—C13	119.60 (11)
C5—C6—H6	120.6	N1—C14—C9	121.18 (12)
N1—C7—O1	121.67 (11)	C13—C14—C9	119.22 (12)
N1—C7—C8	123.96 (12)		
			
C7—O1—C1—C6	−117.62 (13)	O1—C7—C8—N2	−178.58 (11)
C7—O1—C1—C2	66.18 (16)	C8—N2—C9—C10	179.57 (11)
C6—C1—C2—C3	0.5 (2)	C8—N2—C9—C14	−0.26 (17)
O1—C1—C2—C3	176.56 (12)	N2—C9—C10—C11	−179.85 (12)
C1—C2—C3—C4	−0.4 (2)	C14—C9—C10—C11	−0.03 (19)
C2—C3—C4—C5	0.0 (2)	C9—C10—C11—C12	−0.3 (2)
C3—C4—C5—C6	0.3 (2)	C10—C11—C12—C13	0.0 (2)
C2—C1—C6—C5	−0.2 (2)	C11—C12—C13—C14	0.6 (2)
O1—C1—C6—C5	−176.37 (11)	C7—N1—C14—C13	179.44 (12)
C4—C5—C6—C1	−0.2 (2)	C7—N1—C14—C9	−1.19 (17)
C14—N1—C7—O1	179.35 (11)	C12—C13—C14—N1	178.41 (11)
C14—N1—C7—C8	0.49 (18)	C12—C13—C14—C9	−0.98 (19)
C1—O1—C7—N1	−1.81 (18)	N2—C9—C14—N1	1.12 (18)
C1—O1—C7—C8	177.15 (10)	C10—C9—C14—N1	−178.70 (11)
C9—N2—C8—C7	−0.45 (18)	N2—C9—C14—C13	−179.50 (12)
N1—C7—C8—N2	0.3 (2)	C10—C9—C14—C13	0.68 (18)
